# Case of Pneuma-Thorax in a Medical Gentleman; with an Account of an Operation Performed for Its Relief; the Effects of the Operation; the Examination after Death, and Remarks on Pneuma-Thorax Generally

**Published:** 1829-04-01

**Authors:** 


### PERISCOPE.

### XIII.

### CASE OF PNEUMO-THORAX IN A MEDICAL GENTLEMAN; WITH AN ACCOUNT OF AN OPERATION PERFORMED FOR HIS RELIEF; THE EFFECTS OF THE OPERATION; THE APPEARANCES ON DISSECTION; AND REMARKS ON PNEUMO-THORAX GENERALLY.

One of the most remarkable cases of this disease on record, has recently occurred; and being in the person of a medical practitioner, excited intense interest among a very large circle of physicians and surgeons, who were visitors or attendants on one of their brethren. The case was exceedingly important in many points of view, and was watched and examined, both before and after death, by a greater number of medical men than were ever before seen round the bed of a private patient. The diagnosis and event were looked to by a large assemblage of practitioners, as decisive of the merits or fallacy of auscultation and percussion—and the reputation of one man, at least, was at stake. We shall now proceed to the particulars.

Mr. CORNISH, SURGEON, residing in Milner-place, near the Cobourg Theatre, and aged about 25 or 26 years, became affected with dyspnœa and symptoms of thoracic inflammation, about the latter end of November or beginning of December last, which he neglected for many days, and continued to pursue his avocations in the three branches of the profession. About the 15th or 16th of the same month he was accidentally seen by Mr. Cooke, an intelligent practitioner of Bridge-street, Lambeth, who strenuously recommended sanguineous depletion, confinement to the house, and the other items of the antiphlogistic treatment. It was with difficulty he could be persuaded to take to his room; but he was too ill to go on longer with his practice.

On the 19th or 20th of December, Dr. Johnson was requested to see Mr. Cornish, and found him in the following condition. The patient was of the scrofulous constitution. He was lying on a sofa, on his right side, breathing with considerable difficulty, and frequently coughing. The expectoration was scanty and extremely tenacious, but without any purulency. The pulse was 130, sharp and wiry—skin not very hot nor dry—tongue moist, thirst moderate, right cheek flushed, urine high-coloured and scanty. He complained of great difficulty of breathing, had pain in the centre of the chest, and could only lie on the right side. On uncovering the thorax, the muscles of respiration were seen in violent action, but the breathing was principally carried on by the diaphragm. There was no perceptible difference in the size of the two sides of the chest; but a very remarkable difference in the sound emitted on percussion. The *left* side sounded louder than natural—the right sounded considerably duller than natural. On applying the ear to the *left* side, which sounded so well, little or no respiration could be heard. On listening to the *right* side, which sounded so dull, the respiration was very loud, and accompanied with much wheezing. The heart was felt beating rather to the *right* of the middle of the sternum, and no trace of it could be felt in the *left* side.

These phenomena appeared to Dr. Johnson to be very unfavourable; but as inflammatory action was still unequivocal in the case, Dr. J. advised Mr. Cooke, who kindly and zealously attended his afflicted neighbour till the last, to take away more blood, both generally and locally. Digitalis, colchicum, and antimony were also given, in powerful doses, with the view of making an impression on the circulation.

*21st December*. The urgency of the dyspnœa was a little, and but a little relieved, by the depletion. The blood was remarkably buffed and cupped. On examining the chest this day, Dr. Johnson and Mr. Cooke found that the left side was even more sonorous than before—and the respiration there still more indistinct. The pulsation of the heart was rather farther to the right—the right side very dull on percussion, and the respiration very noisy and confused. But a most important feature of the case now attracted attention—namely, the METALLIC TINKLING, (tintement metallique,) which was distinctly audible in the left side of the thorax, not only when the patient coughed or spoke, but even during every inspiration and expiration. Dr. Johnson had now no doubt of the existence of PNEUMO-THORAX, as every person who put the ear to the chest, heard the tinkling as plainly as himself. Upon accurate examination, the left side was found to be very sonorous back almost to the spine, which led to the conclusion that the quantity of serous, purulent, or sero-purulent effusion, was very small in quantity, when compared with the aeriform extravasation. What was now to be done? There were still symptoms of thoracic inflammation present; and to quell these, and promote a free expectoration, every mean that could be devised was put in force. The next five or six days were consumed in the furtherance of these indications, but with no effect in mitigating the difficulty of breathing, which, indeed, gradually increased, the pulse seldom coming under 130 in the minute, with great and distressing jactitation. In the course of the above period, several medical gentlemen saw the patient, and Dr. Walshman was added in daily consultation with Dr. Johnson and Mr. Cooke.

On Monday night, the 29th December, the patient nearly expired from suffocation, and next morning, (Tuesday, the 30th,) Dr. Johnson explained to the patient the nature of the case—namely, that there was an aperture in the left lung, through which air was extravasated into the left pleural cavity, which cavity also contained some fluid, the precise nature of which could not be ascertained It was stated to Mr. Cornish that the increasing collection of air was pressing severely on the right lung—that it had already pushed the heart into the right side of the chest—and that there was no prospect of relief, but from an operation. Dr. Blicke, of Walthamstow, examined the patient on Tuesday morning, with Dr. Johnson, and was so convinced of the existence of pneumo-thorax, as the cause of the dreadful dyspnœa, that he volunteered to perform the operation. Things, however, were not sufficiently ripe for such a step, and Dr. Johnson requested the patient to name a surgeon of eminence to join in the consultation. He named Mr. Lawrence, and Dr. Johnson waited on Mr. L. to request his opinion on the case. Mr. Lawrence, Dr. Walshman, Mr. Cooke, Mr. J. H. Johnson, and some other medical men, met at 3 o'clock on that day. Mr. Lawrence accurately examined the patient. He was lying on his right side, as usual, breathing most laboriously, his countenance sunk, the pulse between 130 and 140, weak and somewhat irregular. The skin was cool and somewhat moist—he had had no sleep for many nights. On laying bare the chest, the action of all the respiratory muscles was painful to behold—and it was evident that but a very small portion of air could be taken in at each inspiration. There was no perceptible difference in the size or shape of the two sides. The *left* sounded hollow throughout almost its whole extent, when Mr. Lawrence struck it—the right side emitted an extremely dull sound. The apex of the heart was now beating rather to the right of the right nipple. When Mr. Lawrence applied his ear to the left side of the thorax, he distinctly heard the metallic tinkling, as did every one of the medical gentlemen then present.* The respiration was loud and rattling in the right lung, and the expectoration muco-purulent, with streaks of blood, and many black particles.

On retiring to consult, it was the opinion, not only of Mr. Lawrence, but of all the other attendants, that Mr. Cornish was so near death as to render any operation hazardous, if not unavailing. Indeed it was believed that the patient would most likely expire during such an operation as was contemplated. Mr. Lawrence, however, candidly avowed that he was satisfied of the existence of PNEUMO-THORAX, both from the confidence of Dr. Johnson's diagnosis, and from the phenomena which he had himself observed, during the examination by percussion and auscultation. He also stated it as his opinion that, under more favourable circumstances, and with the same kind of phenomena present, the operation of paracentesis thoracis would be warrantable, as the only probable mean of affording relief, whether temporary or permanent, from the difficulty of breathing resulting from the pressure of air and other fluid extravasated in the cavity of the pleura. An anodyne was prescribed—the gentlemen separated without any resolution to meet again, as Mr. Cornish appeared to be dying, and the unfortunate patient himself expressed the most poignant disappointment that no operation was undertaken for his relief.

* Some of the medical gentlemen present, and particularly Mr. J. H. Johnson, compared the METALLIC TINKLING to the sounds emitted by a musical snuff-box—and this, in reality, is a more familiar, as well as a more exact similitude than that which Laennec has employed.

On that day, Dr. Johnson accidentally met with Dr. Ballingall, of Edinburgh, Mr. Pecchioli, of Florence, and Mr. Guthrie. To these gentlemen he related the melancholy case of Mr. Cornish, and they having expressed a wish to see him, if yet alive, Dr. Johnson solicited them to visit the patient with him. They repaired to Mr. Cornish's house at 10 o'clock at night, and found the patient nearly in the same state of distress as he was in at 3 o'clock, when Mr. Lawrence and Dr. Johnson left him. The gentlemen above-mentioned recognized the ausculatic phenomena which have been already detailed, and, in consequence of a most urgent solicitation, not only from the patient, but from his sisters and several relations, Mr. Guthrie agreed, in deliberate consultation with Dr. Ballingall, Mr. Pecchioli, Mr. Cooke, and Dr. Johnson, to perform the operation of paracentesis thoracis, as the only measure that offered even temporary relief from the dreadful state of suffocation to which the unfortunate patient was reduced. The danger of the case was not concealed from Mr. Cornish himself, nor from any of his friends—nor was any sanguine expectation held out of recovery, but only of relief. It was stated that the operation was neither painful nor dangerous, and that it afforded the only probable chance of life that remained. The patient and friends ardently urged the operation.

An incision was made in the anterior lateral part of the left side of the chest, between the sixth and seventh ribs, and the pleura cautiously opened with the scalpel. At that instant a rush of air issued forth, with a loud hissing noise, and strong enough to extinguish several candles, had they been near the orifice. The relief was almost instantaneous. The patient turned on his back, and breathed with comparative freedom, expressing the highest sense of gratitude for the operation. He was turned round on the left side; but no fluid came from the wound. A piece of linen was placed over the orifice, and the medical gentlemen retired. The relief continued for some hours, and then the difficulty of breathing returned to a certain extent.*

* On returning home, at midnight, Dr. Johnson wrote a note to Mr. Law-

*Wednesday* 31*st*. Mr. Guthrie, Mr. Cooke, Dr. Johnson, and several others, visited the patient at half-past 12 o'clock, and found him labouring under a considerable degree of dyspnœa, though not near so much as before the operation. It was found, on examination, that the wound was closed. The left side sounded nearly as sonorous as ever, and the TINTEMENT METALLIQUE was perfectly audible. A director was introduced into the wound, and a rush of air instantly escaped, with immediate relief, as in the first operation. A probe-pointed bistoury was passed in, and the opening in the pleura extended to the size of half an inch. The pulse had fallen to 120, the countenance was good—skin moist—expectoration more copious, and muco-purulent. On examination of the leftside immediately after the escape of the air, no "TINTEMENT METALLIQUE" could be heard by any of the medical gentlemen. The patient took nourishment this day, and was seen by several medical practitioners. In the evening, when Dr. Johnson visited him, the patient was not so well, and a probe was again introduced, when air escaped with some noise. Twenty drops of laudanum were given in a saline draught, and the patient was left.

*Thursday*, 1*st Jan*. 1829. On visiting Mr. Cornish this day, the medical attendants were agreeably surprised to find that the patient had had several hours of tranquil sleep—and that for the first time during some weeks—that his breathing had been easy—the expectoration more copious, and inclining to purulency—the pulse reduced in frequency, and more expanded—the appetite good. He got out of bed this day, without assistance, went to the commode, and had a natural motion. Mr. Lawrence saw the patient, and pronounced him greatly relieved. On examining the wound this day, a canula was pushed in, and a taper was held near it. During inspiration the canula was closed with the finger, so that no air could enter the chest—and during expiration, the finger was removed from the canula, when a rush of air always escaped. This was continued until no doubt could remain as to the fact, that part of the air drawn in by the mouth was thrown out of the wound at each expiration. This phenomenon, and especially the large quantity of air thrown out at each expiratory movement, proved that a considerable aperture of communication existed between the bronchia and the cavity of the pleura. This circumstance greatly lessened the hopes of recovery. It was found that, since the operation, the apex of the heart beat about an inch and a half, or two inches nearer the central line of the thorax than before. The pulsation was still, however, to the right of this line. The patient continued comfortable through the day; but Mr. Cooke was called up in the night, and found him greatly oppressed. The canula was re-introduced, and some relief followed, the wound being covered with a piece of gauze.

*Friday*, *Jan*. 2*d*, 1829. It was but too evident this morning that the unfortunate patient was sinking. He had a strong convulsion early to-day, and about one o'clock he expired.

POST-MORTEM EXAMINATION.

Mr. Cornish being of the Hebrew religion, great difficulties lay in the way of an examination *post mortem;* but the friends and relatives of the deceased evinced much liberality, and leave was ultimately attained for dissection, though such a process was almost unprecedented among the Hebrew brethren. There were present at the examination a very great number of medical men, among whom were Dr. Walshman, Dr. Hodgkin, (who officiated as dissector,) Dr. George

---

rence, apologizing for the apparent breach of etiquette, and inviting Mr. L. to see the patient next morning, stating what had been done. The following is Mr. Lawrence's reply:—

"My dear Sir,

I am much obliged by your letter, and regret that my engagements for the day prevent me from having the pleasure of meeting yourself and Mr. Guthrie. Whatever may be the final result, the relief obtained by the patient from such urgent distress, not only proves the correctness of your diagnosis, but also fully justifies the operation.

Your's,

WM. LAWRENCE."

*Whitehall Place*,

31*st Dec*. 1828.

Gregory, Mr. Guthrie, Mr. North, Mr. Cooke, Mr. Cooke, junior, Mr. Canstatt, Mr. Way, Mr. J. H. Johnson, and others. Previously to the examination, Mr. C———, a surgeon of the Hebrew religion, who had frequently visited the deceased during his illness, demanded of Dr. Johnson what were the morbid appearances which he expected to find? Although this was a question which it would not be always very charitable to ask, before a dissection, yet Dr. Johnson did not decline the answer, which was made in the presence, not only of the above medical gentlemen, but of a number of the patient's friends. "*The disease was pronounced to be* PNEUMO-THORAX—*and the morbid appearances would be, a collection of air and some other fluid in the left side of the chest—collapse of the corresponding lung—aperture in the lung capable of giving free vent to air from the lung to the cavity of the pleura—displacement of the heart—probably tubercles in the right lung.*"

Dr. Hodgkin then opened the body. On raising the sternum, the heart was found rather to the right of the median line of the chest. The left lung was collapsed to one-fifth of its natural dimensions. The vacant space was filled with air, and about fourteen ounces of turbid serous fluid. The pleura costalis and pulmonalis presented marks of inflammation, of a few weeks' standing—viz. some thin false membranes that were easily separated by scraping with the scalpel. There were no marks of any more recent pleuritis, even in the vicinity of the wound, there being only a slight ecchymosis between the pleura and subjacent cellular tissue, for the space of a few lines around the incision. A tube was inserted into the trachea, and air blown into the lungs. The left lung expanded to a certain extent, and air was heard to bubble out. The lung was then carefully removed, and an aperture was immediately recognized at the division, or cleft between the two lobes. The tube was inserted into the bronchus leading to the left lung, and Dr. Johnson blew in air. It rushed forth at the aperture, and extinguished a taper that was held near it. The aperture itself was then more accurately examined. It was circular, and capable of admitting a crow-quill. It was evidently fistulous, and of several weeks' standing. It was found to communicate with a very small excavation formed by the softening down of some tuberculous matter—and into this small excavation a bronchial tube was seen to enter. Thus the communication between the trachea and the cavity of the chest was distinctly traced through a bronchial ramification, a very small tubercular excavation situated on the very surface of the lung, and an aperture through the pleura pulmonalis. The left lung presented some trifling tuberculation, but was not materially diseased.

The right lung was much more tuberculated; but the tubercles were principally in a quiescent state. There was no other disease in the chest. Dr. Hodgkin formally declared, that every iota of the diagnosis was verified by dissection, and every individual present agreed in this declaration.

It may now be interesting to endeavour to trace the course, or the order of succession in the foregoing morbid appearances. The tuberculation of the lungs was of old standing—probably of many years' duration. The softening down of the tuberculous matter on the surface of the left lung must have preceded the formation of the aperture and escape of air into the cavity of the pleura. The most interesting question is, at what period did the pneumo-thorax commence? It appeared that Mr. Cornish dated his last illness to his being called out to a case of midwifery in the latter end of November or beginning of December, during a cold foggy night, and to his running hard and putting himself out of breath. From that moment he began to find his respiration incommoded, and the symptoms of thoracic inflammation commence. In illustration and support of this position, it may be useful to revert to a case published in the 18th number of the Medico-Chirurgical Review, (p. 566,) from the Hôpital St. Antoine. The patient was a young woman, who, six weeks prior to her admission into the hospital, felt something give way, while in the act of coughing, followed by difficulty of breathing, palpitation of the heart, and some spitting of blood. On examination at the hospital, (six weeks after the above accident,) the respiration was found to be short and embarrassed—the left side of the chest was remarkably sonorous on percussion—while the right side sounded dull. The breathing was loudly heard in the right side of the chest—no respiration, but the "METALLIC TINKLING," in the left side of the thorax. The heart pulsated in the right side. M. Rayer pronounced the existence of PNEUMO-THORAX. Nothing was done, and the unhappy patient lingered in a dreadful state of suffocation for more than a fortnight, when death put a period to her sufferings. On dissection, the left lung was found compressed to a very small size—the cavity of the pleura was filled with air—the heart was pushed over into the right side—there was an aperture in the left lung—and tubercles in both lungs.

Here then is a case precisely similar, in every living symptom and *post-mortem* appearance, to that of Mr. Cornish. The pneumo-thorax must here have existed at least eight weeks before death. In Mr. Cornish's case, there were symptoms of pneumo-thorax on the 19th or 20th of December, when Dr. Johnson first saw him, combined with thoracic inflammation. It is therefore justifiable to infer, that the rupture of the pleura took place on the night when Mr. Cornish ran to his patient—and that it was then, or soon after that period, that the pleuritis commenced. At all events, the difficulty of breathing was dated from this time, and there is no reason to deny that a rupture of the pleura pulmonalis and extravasation of air into the cavity of the pleura should induce inflammation of the lining membrane of the chest. Dr. Hodgkin stated it as his opinion that the marks of inflammation in the pleura evinced a pleuritis of from three to five weeks' duration.*

The next thing to be accounted for is the serous effusion in the left side. Every pleuritic inflammation is accompanied by more or less of serous effusion; but the sonorous state of the whole of the left side, and the circumstance that no fluid escaped on turning the patient quite over on the left side, as well as the fact that he lay during his distress on his right side in preference, induce the belief that, by far the greater portion of the fluid found after death, was effused during the last few hours of existence—in the agonies of death—or after life had been extinguished.

In respect to the operation, it may be proper to observe that it was not the hasty project of the moment, but the result of deliberate reflection. In the July number of the Medico-Chirurgical Review, after stating the case of M. Rayer's, and also another somewhat similar, Dr. Johnson made the following observation.

"Now we must acknowledge that the two individuals above-mentioned, did not present, on dissection, any organic disease incompatible with life—or, at least, with a prolongation of life. They evidently died in consequence of the effusion of air into the cavity of the chest compressing the lungs, displacing the heart, and impeding the vital function of respiration. They were, in fact, placed, as nearly as possible, in the same situation as individuals who had been wounded in the lungs by a small sword, or by the fractured ends of ribs. Why then hesitate to make an opening through the intercostal spaces, and extricate the effused air? Every time the patient breathed, some air was forced out into the cavity of the pleura, and it could not get back through a valvular opening. The obvious remedy was a free exit from the chest till the channel of communication closed."—MED.-CHIR. REV. No. XVIII. p. 568.

Laennec, who first described pneumothorax systematically, and who must have seen many fatal cases of it, does not appear ever to have witnessed or practised an operation for its relief, except in one instance, where the auscultic indications

* That the effusion of air preceded the pleuritis, is proved almost to a demonstration, by the existence of false membranes on both pleura pulmonalis and pleura costalis, without the slightest trace of adhesion between any portions of these membranes. The intervening air prevented such adhesions. This non-adhesion could not have been the consequence of the serous effusion. The phenomena shewed that the serous effusion was very trifling; and had it existed during life, even to the extent found after death, it could not have prevented adhesions about the upper and anterior parts of the chest. This freedom from adhesions, then, resulted from effused air, which would diffuse itself equally over the whole internal surface of the left side.

of pneumo-thorax being very evident—a sense of fluctuation perceptible—and the oppression rapidly increasing, an incision was made between the fifth and sixth rib, near their middle. M. Laennec's words are—"no matter flowed, although the passage of air by the wound during respiration proved the penetration of the chest by the incision." The patient died in four hours after the operation. On puncturing the side, near the junction of the third rib with its cartilage, a large quantity of fetid gas made its escape. On making another incision about the middle of the fourth intercostal space, a large quantity of pus flowed out, of an intolerably gangrenous fetor. The reason of this want of success in evacuating the pus, was adhesions in different parts of that side. The case was evidently one of empyema combined with pneumo-thorax, and therefore not parallel with Mr. Cornish's.

M. Laennec, in speaking of the operation for pure pneumo-thorax, believes that it has sometimes been performed *by accident*, namely, where the chest has been opened for empyema or hydrothorax, and only air has escaped. The above authority gives it as his opinion that the operation offers a far greater chance of success in simple pneumo-thorax, (such as Mr. Cornish's case,) than in cases complicated with empyema, phthisis, or other serious lesion. In the case forming the subject of this paper, there was no disease in the chest incompatible with life. There was no organ irrecoverably altered in structure, excepting perhaps the fistulous aperture in the left lung—and therefore the patient died from impeded function of that portion of lung which was not collapsed, viz. the right lung. The main cause of the impeded function in the right side, was pressure of the air collected in the opposite side—and nothing but an operation could relieve this. Laennec distinctly states it as his opinion, that the operation should be performed *whenever the life of the patient is threatened by suffocation.* No one will deny that Mr. Cornish was threatened with this dreadful death.

The next question is this:—Have we any means of ascertaining PNEUMO-THORAX, and, consequently, of deciding on the propriety of the operation, except by auscultation and percussion? None. The difficulty of breathing might arise from various causes; the position of the heart might be congenital—succussion, which was tried, gave no evidence of fluid, because there was so little; and could not afford any indication of air.

Neither would percussion *alone* enable us to fix the nature of the case, and determine on an operation. It is true, the left side was more sonorous than the right—a phenomenon which, without auscultation, would lead to the supposition, that the right side was the principal seat of disease. But who would venture on such an operation, merely because one side was more sonorous than the other? When auscultation was added to percussion, and when these two means of diagnosis were brought in aid of symptomatology, then all doubt was removed. When it became evident that the left side, which sounded best, afforded little or no respiratory murmur—when the respiration was heard loud in the right side, which sounded dull—and when the metallic tinkling was constantly heard in the left side, there was a degree of certainty given to the diagnosis, and of confidence to the hand of the operator, which the most accurate symptomatology could not possibly afford.*

There is every reason to believe that spontaneous cures of this dangerous disease have taken place. Laennec, indeed, records one or two instances, where life was preserved after the occurrence of pneumo-thorax—but, as air is sometimes generated without an aperture in the lung, there is no certainty that this last circumstance actually obtained in such fortunate cases. The way in which Nature performs the cure is this:—When a communication is made between the lung and the cavity of the pleura, air is poured out, with more or less rapidity, and the lung being compressed, gradually collapses, if no adhesions exist, till it can collapse no more. In this last state, it

* In Dr. Davy's case, recorded in the Philosophical Transactions for 1823, and which will be presently noticed, the left side was bulged out, tense, and evidently tympanitic. This bulging out removed all uncertainty, and rendered the diagnosis much more easy than in Mr. Cornish's case.—ED.

is in the most favourable condition for the healing of the aperture. If the opening heals, the air will be absorbed and the patient will recover. If it does not heal, the accumulation will go on increasing—the opposite lung will become compressed—and death will result from the interruption of the respiratory function.

The object of the operation is to prevent suffocation from this accumulation, and to leave the lung in a quiescent state, favourable for the healing of the aperture.

The mode of operating in these cases, and the management of the wound afterwards, are circumstances of the highest importance for consideration, as on these the event will materially depend. It was supposed, in Mr. Cornish's case, that, as there was no evidence of any material quantity of fluid in the chest, a small aperture would be sufficient, in the first instance, for the discharge of the air. And so it was. But as the air accumulated again, with corresponding increase of dyspnœa, the wound was not only re-opened, and the air discharged, but it (the wound) was enlarged, by means of a probe-pointed bistoury. It was expected that an opening between the ribs would give sufficient exit to air, whenever it accumulated to an extent capable of making much pressure on the opposite lung—and that thus the danger of kindling up a new inflammation in the cavity of the pleura by the constant presence of a canula, would be avoided.

Mr. Guthrie, whose experience in wounds of the chest has been great, stated, on performing the operation, that the wound must be kept open, if the ulcerated or fistulous aperture (which was expected) should be found to exist in the the lung—and that, although air alone might be evacuated in the first instance, the inevitable consequence of such a wound would be, (between the third and fifth day) the effusion of a larger quantity of fluid than might be expected by persons unaccustomed to such injuries—which fluid must be allowed a ready exit as soon as its presence was ascertained. The opening in the pleura, of half an inch in extent, only allowed the air to pass in and out freely for a short period after each examination; and a round-pointed tube was introduced, for the purpose of giving it a free exit whenever the breathing became oppressed. A due consideration of the subject leads to the conclusion, that the opening should be made sufficiently large to allow free egress to the air at all times;—or a small canula must be retained in the opening for the purpose, long enough to pass beyond the pleura, but scarcely doing more than entering the cavity of the chest, and which can be removed as soon as the wound has become sufficiently pervious for both air and other fluids, the products of the inflammation consequent on the operation.

The quantity of compressed air in the chest may always be judged of by the sound communicated to the ear, when closely applied to the side. The "TINTEMENT METALLIQUE" is as loud as the sound of a small musical bell struck by the nail, when the quantity is considerable, and degenerates into a mere metallic murmur, when the PRESSURE of the air is taken off by a free communication with the external atmosphere.

In this country, there is but one other case on record, where the operation was performed for pure pneuma-thorax, and the operation was successful. The circumstances of the case, however, were different, and the diagnosis was infinitely more easy in the one than in the other, as will be seen. The case is recorded in the Philosophical Transactions for 1823, by Dr. Davy.

A soldier was sent home invalided from the West Indies, for hæmoptysis, *which had succeeded a severe fall on the left side of the chest, received* 18 *months previously*. He was admitted into the Military Hospital of Chatham on the 9th May, 1823. On the morning of the 13th, after a violent fit of coughing, symptoms of pneuma-thorax came on suddenly, and continued increasing till the 21st. "The most prominent of these symptoms were, (to quote the words of Dr. Davy) a feeling of extreme tightness about the chest and abdomen—rapid and difficult inspirations, between 30 and 40 in the minute—great anxiety of countenance and agitation of mind, accompanied by a small pulse of 130—cold sweats frequently breaking out on the neck and face—considerable prostration of strength. On examining the chest, *the left side was found more protuberant, and, in all its dimensions, larger than the right*; it was tense, and, on percussion, sounded remarkably hollow and tympanitic, giving the idea of its being distended with air. The heart was found beating under the right mamilla."

Under these circumstances it was resolved, in consultation, to tap the chest, which was accordingly done in the following manner. A trocar was attached to an empty bladder, and the parietes of the chest punctured between the eighth and ninth ribs,* the integuments and intercostal muscles being previously divided with a scalpel. A little air only rushed out, and, as it was concluded, from the symptoms continuing, that its escape had been prevented by adhesions of the pleura at the point which had been perforated, the operation was repeated next day. The puncture was now made just below the left papilla, when on withdrawing the stilet into the bladder, a large quantity of air rushed out and distended it. The bladder was now separated. Air continued to rush from the chest for several seconds, "as if from a blow-pipe." When this ceased, and when the air began to pass inwards during inspiration, the canula was withdrawn, and the wound healed. The relief to the patient was sudden and great, and he continued to improve till the 17th of June, when the account closed. No farther history of the case appears to have been published. The heart was still felt beating in the right side, and "the fluctuation of a fluid was perceptible in the left."

It will be obvious that this was a case much more easily discriminated than that of Mr. Cornish. The eye alone shewed that the left side was distended with something, and percussion shewed that it was chiefly air. The operation was as plainly indicated as in empyema, and auscultation does not appear to have entered into the means of diagnosis.

It differed in another very important respect from the case of Mr. Cornish. When the air was evacuated, the wound was closed, and no more air became accumulated. This proves one or other of the following circumstances, viz. either the original aperture in the lungs, through which the air had passed out, became closed before the operation—or, what is not unlikely, the air was generated in the cavity of the pleura after the effusion of some purulent or sanguineous discharge, and when once evacuated, was no longer formed. In either case, the circumstances were much more favourable for recovery than in Mr. Cornish's case, where the aperture in the lung had become actually fistulous, and capable of affording a stream of air sufficient to extinguish a candle. It is worthy of remark, that a general adhesion of the pleura costalis and pulmonalis, from preceding pleuritis, would be a complete safeguard against pneuma-thorax—and that partial adhesions would render the disease comparatively harmless, by preventing such accumulation of air in one side of the chest, as would much compress the lung of the other. This was evidently the case in Dr. Davy's patient.

From the facts already stated, it might be fairly inferred that, had the operation been performed at a much earlier period in Mr. Cornish's case—in short, when the pneuma-thorax was first ascertained, and when the difficulty of breathing was urgent—and had the aperture in the lung healed, as it probably would have done, his life might possibly have been saved.

The case, at all events, must form a precedent for the safety of the operation —for the relief, whether temporary or permanent, which it affords to one of the most dreadful kinds of human suffering, suffocation—and for the certainty of diagnosis afforded by auscultation.* It has

* This was certainly rather low, as the attachments of the diaphragm must have been touched in the operation.

* Some doubt has been recently attempted to be thrown on the diagnostic mark, the "TINTEMENT METALLIQUE," by a case which occurred in LA CHARITÉ. A patient came in with symptoms of phthisis; but, on examining the chest, one side was found bulged out, and but very little respiration could there be heard. The "tintement metallique," however, was very audible, and that side sounded much duller than the other. The patient died without any operation, and a considerable quantity of pus was found in the affected side, without any air. On accurate examination of the collapsed lung, a fistulous aperture was found, but so small that they could only force some already formed a precedent of another kind—permission to examine the Hebrew dead. Such was the intense interest excited among a large circle of the patient's Jewish friends, that a law hitherto considered as almost insuperable, was broken through, in order to determine whether the operation had been fully justified, not only by the relief obtained while living, but by the evidence to be drawn from the corpse.

It may naturally be asked, how would Nature effect a cure in such a case, provided the external aperture was kept open till the pulmonary aperture healed? This may be answered by an appeal to facts. In cases of very severe pleuritis, there is a considerable effusion into that side of the chest, with a proportionate compression of the lung. The inflammation having ceased, the process of absorption commences, and as this goes on, the lung gradually expands on one hand, and the chest contracts on the other. The thinner parts of the effused fluid being at length entirely absorbed, the lung and the pleura costalis become agglutinated together by a false membrane, with more or less falling in of that side. A somewhat similar process would take place in pneuma-thorax,—the object of the operation being to prevent suffocation from oppression of the opposite lung.

It is to be hoped that this case has thrown no discredit on the science of either medicine or surgery—and that it has not tended to lessen the confidence of the extra-professional public in the certainty or the resources of an art, which is almost as often called upon to mitigate the sufferings of the dying as to arrest the hand of death.

---

bubbles of air, and not a stream through the aperture. There is great reason to suspect that some fallacy has crept into the account of the dissection, and that, as there was a fistulous aperture, there was also effused air to account for the "metallic tinkling." At all events, the want of SONOREITÉ in that side, and the bulging out of the intercostal spaces, could leave no doubt of the existence of empyema, and the operation was justifiable on that account alone.

### XIV.

### VISCERAL NEURALGIA, IN A MEDICAL GENTLEMAN.

We have drawn the attention of our brethren, on several occasions, to this distressing malady—a Proteian enemy which often baffles the most strenuous efforts of medicine. It may be proper to premise that the sufferer, in the present instance, is a young medical gentleman in extensive practice in Wiltshire. About five years ago, and almost immediately after finishing his studies in London, he was attacked with occasional pains in the epigastrium, which, however, were slight, and not productive of much inconvenience. Having engaged in very laborious duties, as an assistant, in Devonshire, the complaint disappeared; but, on sojourning for a time, near Windsor, the pain returned. The gentleman next settled in a pleasant and healthy town in Wiltshire, and there he has suffered, and continues to suffer, under a malady which shall be described in his own words.

"It is now three years since I commenced practice, and during nearly this whole period, I have almost incessantly suffered the torment of the Proteian malady. The following is the manner of attack:—The pain generally commences, as well as I can judge, about the situation of the pyloric orifice, or duodenum, and I can best describe its nature by saying, that the feeling is similar to that which I should expect to feel from being incised with a cutting instrument, in a straight line, to the extent of three or four inches In a few minutes, the pain radiates from this centre to almost every part of the abdomen, and also to the chest and back. I feel them more severely at the pit of the stomach, and in the back, at that part exactly where the duodenum is tied down to the spine. There is a particularly strange feeling of obtuse pain felt about the region of the heart, which I shall describe best in saying, that it feels as if a hand had fixed upon it, and was gently compressing it. There is also, invariably, a good deal of pain and uneasiness in the testes, and in the course of the spermatic chord. Frequently I feel tightness on the chest, have occasionally sore throat, and cough, and a sense of constriction about the pharynx and œsophagus. My pulse seldom exceeds 70, or 75, and the only deviation I can perceive in it from the healthy character, is, that it feels somewhat contracted. My tongue, in the morning, is thickly coated with a tenacious fur, sometimes of a white, and at others of a yellow colour, and at times I have observed it more so on the right side, and indeed it appears at times as if there was a distinct linear division from the base to the tip, the right side being much more loaded than the left. There is no redness, either at the tip or sides. There is a considerable degree of cutaneous irritation, and a pricking sensation frequently over the whole body. My hands and feet are always cold or nearly so, and the skin looks loose and puckered. I am generally overwhelmed with lassitude, and have very little spirit for those active exertions which a wide and extensive practice requires. There is at present that extreme degree of irritability of the stomach, that severe pain is excited by food and drink of almost any kind, however small in quantity, or unirritating in quality. There generally prevails great constipation of the bowels, and the motions are seldom of a natural colour, being generally very dark, sometimes of a greenish hue, and often nearly white. I cannot say that my pains are increased by pressure; indeed I may rather term the sensation I feel on kneading myself in various parts of the abdomen, an impatience of pressure rather than absolute pain. I have latterly found horse exercise to seriously exasperate my ailments; and occasionally my pains are so severe, that the mere act of putting the foot to the ground in walking excited a painful concussion of the whole system. *My complaint, at times, distresses me most at night, at other times, in the day time; and it is a curious fact, that frequently for weeks together, the pain attacks me at certain hours of the day, and but little at night; then again the reverse takes place—I have most pain in the night, and comparatively little in the day time.*

The medicines I have resorted to consist of those which I have thought best adapted to lessen irritability, such as prussic acid, hyosciamus, stramonium, &c. On one occasion I took, by the mistake of one of my pupils, 2 grains of belladonna, for 1; and it is a very curious fact, that after having suffered severely from the peculiar effects of this powerful narcotic, I had no return of pain for two months! The effects produced, however, deterred me from its continuance. The prussic acid seemed to exasperate the pain—stramonium, nor hyosciamus did nothing for me, nor do large doses of opium. But I will now mention a remedy, but for which I think I must for a long time since have yielded to the enemy. It is magnesia, which I take invariably on the accession of the paroxysm; and it does not appear to be of any consequence whether I take it in a calcined or the common form. Another article of diet I am still more indebted to you; *and that is milk. Either of these articles acts on me like a charm—in less than ten minutes after swallowing either, I am perfectly free from pain for three or four hours. To milk I give the preference on two accounts; first because no harm can accrue from it; next because it relieves me more certainly.* Now the conclusion I draw from this fact is, that the pains I feel are occasioned by vitiated secretions; that magnesia absorbs these secretions, and milk obtunds their acrimony. I consider then that these depraved juices act on the mucous membrane of the stomach precisely in the same way as do the corrosive and irritating poisons, inasmuch as they are relieved by the same remedies. I have also occasionally tried sugar and water, and albumen, and both of these produce relief, though not to the extent of the milk and magnesia. These are curious facts, I think; and I hope you will not for one moment consider that I am influenced either by imagination or prejudice in detailing them, but that you will consider them as the result of personal experience. Blue pill has never appeared to relieve me—nor rhubarb—nor, in fact, any tonic; at least of the vegetable kind, but produced, as I should have expected *à priori*, increased pain and irritation.

Now, my good Sir, I hope you will feel for a labourer in the same vineyard, and give me the best advice you possibly can in my case; for I am assured you will feel for me."

Our readers will perhaps recollect that, in a former number of this Journal, we mentioned the remarkable effect of milk or cream, in allaying the distressing sensations of cardialgia. The reason why we have taken the liberty of publishing the foregoing extract, which we are sure our patient will excuse, is to state that, more than two or three instances have since occurred, where the milk had a similar effect. We should be inclined to exhibit the magnesia in milk. As to the distressing complaint above-described, we are convinced that it is a neuralgia, and of the intermittent or remittent character. The passage which we have marked in Italics proves this. But there is no doubt that some poisonous, corrosive, or highly irritating secretion takes place at the time, which aggravates tenfold the pain in the epigastrium. We shall probably give the sequel of this interesting case on a future occasion.

